# Combined Transcriptome and Metabolome Analysis of Smooth Muscle of Myostatin Knockout Cattle

**DOI:** 10.3390/ijms24098120

**Published:** 2023-05-01

**Authors:** Mingjuan Gu, Song Wang, Anqi Di, Di Wu, Chao Hai, Xuefei Liu, Chunling Bai, Guanghua Su, Lei Yang, Guangpeng Li

**Affiliations:** 1 State Key Laboratory of Reproductive Regulation and Breeding of Grassland Livestock, College of Life Science, Inner Mongolia University, Hohhot 010021, China; gmj0119@yeah.net (M.G.); wangsong199852@163.com (S.W.); anqi_di@126.com (A.D.); wudi2020imu@163.com (D.W.); h15248037201@163.com (C.H.); liuxuefei1006@126.com (X.L.); chunling1980_0@163.com (C.B.); suguanghua0707@163.com (G.S.); 2 College of Animal Science, Inner Mongolia Agricultural University, Hohhot 010018, China; 3 College of Life Science, Northeast Agricultural University, Harbin 150030, China

**Keywords:** myostatin, knockout, transcriptomics, metabolomics, combined analysis, cattle, smooth muscle

## Abstract

Myostatin (MSTN), a growth and differentiation factor, plays an important role in regulating skeletal muscle growth and development. MSTN knockout (MSTN-KO) leads to skeletal muscle hypertrophy and regulates metabolic homeostasis. Moreover, MSTN is also detected in smooth muscle. However, the effect of MSTN-KO on smooth muscle has not yet been reported. In this study, combined metabolome and transcriptome analyses were performed to investigate the metabolic and transcriptional profiling in esophageal smooth muscles of MSTN-KO Chinese Luxi Yellow cattle (n = 5, 24 months, average body weight 608.5 ± 17.62 kg) and wild-type (WT) Chinese Luxi Yellow cattle (n = 5, 24 months, average body weight 528.25 ± 11.03 kg). The transcriptome was sequenced using the Illumina Novaseq™ 6000 sequence platform. In total, 337 significantly up- and 129 significantly down-regulated genes were detected in the MSTN-KO cattle compared with the WT Chinese Luxi Yellow cattle. Functional enrichment analysis indicated that the DEGs were mainly enriched in 67 signaling pathways, including cell adhesion molecules, tight junction, and the cGMP-PKG signaling pathway. Metabolomics analysis by liquid chromatography-tandem mass spectrometry (LC-MS/MS) identified 130 differential metabolites between the groups, with 56 up-regulated and 74 down-regulated in MSTN knockout cattle compared with WT cattle. Differential metabolites were significantly enriched in 31 pathways, including glycerophospholipid metabolism, histidine metabolism, glutathione metabolism, and purine metabolism. Transcriptome and metabolome were combined to analyze the significant enrichment pathways, and there were three metabolically related pathways, including histidine metabolism, purine metabolism, and arginine and proline metabolism. These results provide important references for in-depth research on the effect of MSTN knockout on smooth muscle.

## 1. Introduction

Myostatin (MSTN), a member of the transforming growth factor superfamily, is a principal negative regulator of skeletal muscle growth and differentiation [1]. Deletion of the MSTN gene induces hypertrophied skeletal muscle fibers, leading to a double-muscle phenotype [2]. Double-muscle phenotypes can be identified in different mammalian species with a spontaneous mutation in the MSTN gene, including cattle, sheep, dogs, and humans [2,3,4,5,6,7]. Targeting MSTN gene editing has emerged as a potential strategy in livestock to improve production performance, and has been reported in several domestic animal species including cattle [8], goats [9,10], sheep [11], pigs [12,13], dogs [14], and rabbits [15]. We previously used the CRISPR/Cas9 system to produce MSTN knockout Chinese Luxi Yellow cattle [16]. In addition to skeletal muscles, MSTN also exists in smooth muscles [17,18]. To date, a large number of MSTN studies have focused on skeletal muscle [1,19,20], but only a few have focused on smooth muscle [21,22,23].

Smooth muscles, such as vascular, respiratory, uterine, and gastrointestinal muscles, belong to nonstriated muscles, which are different from the striated muscles of skeletal and cardiac muscles [24]. Smooth muscle cells (SMCs) can form intertwined and elongated sheets of cells in different organs, or they can appear as individual cells [25]. Smooth muscle tissues have no sarcomeres or striations, but unlike skeletal muscles, contractile apparatuses are controlled and formed by actin and myosin [26]. The contraction of smooth muscle tissues is not voluntarily controlled; it is controlled by hormones and parasympathetic signals released through the autonomic nervous system and locally, such as calcium ions (Ca^2+^) [24]. Studies have shown that MSTN has a regulatory effect on smooth muscles [21,22,23]. MSTN knockout can increase the smooth muscle area of the pig esophagus and penile corpus cavernosum and promote the proliferation of sow uterine smooth muscle cells [21,22,23]. However, the effect of MSTN knockout on bovine esophageal smooth muscle has not been reported.

With the development of omics technology, such as transcriptomics and metabolomics, these have been widely used to fully understand the effects of gene knockout on the body [27,28,29]. Transcriptomic profiling is a powerful tool that identifies gene expression signatures [30]. Metabonomics is a useful tool for identifying metabolites and explaining changes in them in a biological system under different conditions [31].

Therefore, the main aim of this study was to elucidate the effects of MSTN knockout on the smooth muscle transcriptome and metabolome. To complete the aim, we performed transcriptome and metabolome analysis of MSTN knockout bovine esophageal smooth muscles, and combined analysis of transcriptome and metabolome data. This study generated genomic and metabolic resources of MSTN knockout bovine esophageal smooth muscles. It provided an important theoretical basis for further in-depth analysis of the role of MSTN in smooth muscles.

## 2. Results

### 2.1. RNA Sequencing and Identification of Differentially Expressed Genes

**Figure 1:**
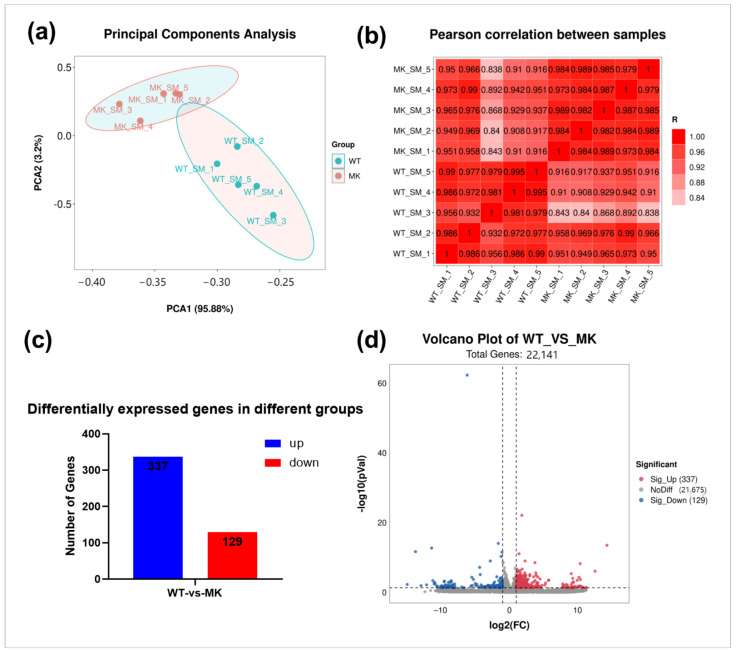
Transcriptome analysis of esophageal smooth muscles from MSTN-KO and WT cattle. (**a**) PCA score plot of transcriptomes. (**b**) Pearson correlation between samples. (**c**) The number of up- and down-regulated differentially expressed genes (DEGs). (**d**) Volcano plot for differential gene expression. (*n* = 5).

The number of raw reads of MSTN-KO and WT bovine esophageal smooth muscle transcriptomes were 262,136,710 and 278,006,580, respectively. After filtering the raw reads for quality control, we obtained 247,222,522 and 262,721,480 high-quality valid reads for MSTN-KO and WT, respectively, with a Q30 base percentage of 97.52% and above (Table 1). Valid reads were aligned with the bovine reference genome (btau4.0) and the comparison efficiency ranged from 96.44% to 97.17% (Table 1). The PCA score plot showed that samples of the inter-group were scattered, and samples of the intra-group were clustered, suggesting good duplication of intra-group and significant differences between inter-group (Figure 1a). The Pearson correlation matrix result was consistent with the analysis of the PCA, which showed high similarity among biological replicates (Figure 1b). Compared with WT cattle, a total of 466 differential genes were identified within MSTN-KO cattle, including 129 up-regulated and 337 down-regulated genes (*p* < 0.05, |log2 FC| > 1), which are shown with bar graphs and a volcano plot (Figure 1c,d). The top 10 differential genes are shown in Table 2.

### 2.2. Enrichment Analysis of Differential Genes

**Figure 2:**
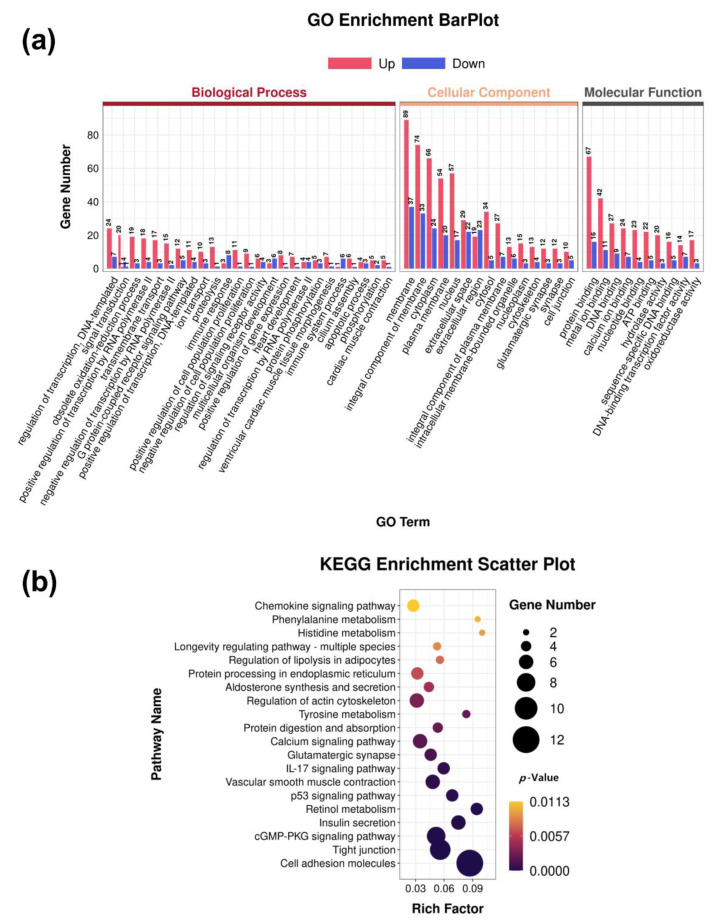
Enrichment analysis of the differentially expressed genes from MSTN-KO and WT cattle. (**a**) Differential expression genes GO enrichment. (**b**) Differential expression genes KEGG enrichment.

To further understand the functions of the DEGs, an enrichment analysis was performed. In total, 52 enriched GO terms were obtained and sorted by Q-value. From the GO terms, 18 enriched GO terms in biological processes (BP), 20 enriched GO terms in cellular component terms (CC), and 14 enriched GO terms enriched in molecular function (MF) were identified (Figure 2a). The top 10 GO terms are shown in Table 3. Furthermore, the enrichment analysis of KEGG pathways included 67 (Q < 0.05) KEGG pathways. DEGs were associated with environmental information processing, organismal systems, human diseases, cellular processes, metabolism, and genetic information processing (Supplementary Table S1). Among them, five pathways were involved in environmental information processing, including cell adhesion molecules, cytokine–cytokine receptor interaction, cGMP-PKG signaling pathway, neuroactive ligand–receptor interaction, and calcium signaling pathway. The five pathways of the phagosome, tight junction, p53 signaling pathway, regulation of actin cytoskeleton, and signaling pathways were involved in cellular processes regulating the pluripotency of stem cells. The six pathways of retinol metabolism, tyrosine metabolism, drug metabolism–cytochrome P450, histidine metabolism, phenylalanine metabolism, and drug metabolism–other enzymes are involved in metabolism (Figure 2b). The top 10 KEGG pathways are shown in Table 4.

### 2.3. Overview of Metabolomic Profiling

**Figure 3:**
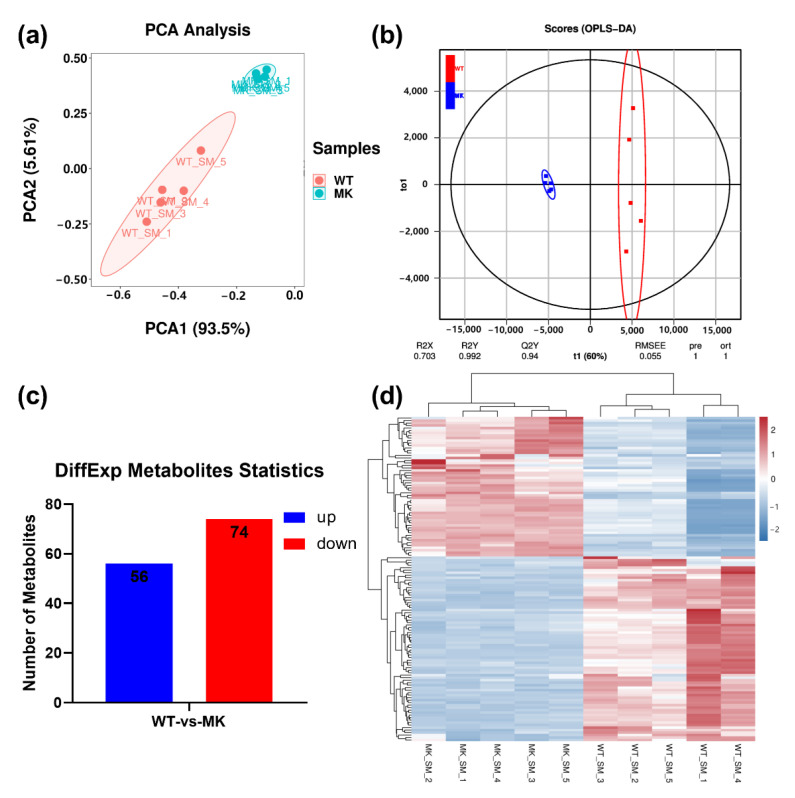
Metabolome analysis of esophageal smooth muscle from MSTN-KO and WT cattle. (**a**) PCA score plot of the metabolome. (**b**) OPLS-DA score plot of all the metabolite features. (**c**) Number of up- and down-regulated differential metabolites. (**d**) Heatmap of the differential metabolites. (*n* = 5).

To investigate the metabolome differences, the metabolites in esophageal smooth muscles of MSTN-KO and WT cattle were analyzed by an LC-MS/MS detection platform and multivariate statistical analysis. PCA, a method of unsupervised multivariate statistical analysis, was performed to determine the overall metabolic differences between groups and the variation within the samples in each group. The results showed that metabolites of MSTN-KO and WT were separated in the score plots, where the first principal component (PC1) was plotted against the second principal component (PC2). PC1 and PC2 represented 93.5% and 5.61% of the total variations, respectively (Figure 3a). Orthogonal partial least squares discrimination analysis (OPLS-DA) was further used to model the metabolite differences between MSTN-KO and WT. As shown in Figure 3b, the two groups were well separated. The OPLS-DA model parameters R2Y and Q2Y were 0.992 and 0.94, suggesting good fitness and predictive ability of the OPLS-DA model. These results indicated significant biochemical differences existed between MSTN-KO and WT cattle.

Based on the OPLS-DA results, metabolites with a fold change of >2 or <0.5 and variable importance in the projection (VIP > 1) were selected for the differential analysis. In total, 130 metabolites were differentially expressed, with 56 up-regulated and 74 down-regulated (Figure 3c,d). The top 10 most differentially expressed metabolites are listed in Table 5. Among them, the up-regulated metabolites included acetyl-DL-carnitine, L-carnitine, and isopropyl tailgate. The down-regulated metabolites included glutamic acid, geranyl citronellol, palmitamide, stearamide, L-glutathione, LysoPC 16:0, and cis-5,8,11,14-eicosatetraenoic acid.

### 2.4. Enrichment Analysis of Differential Metabolites 

**Figure 4:**
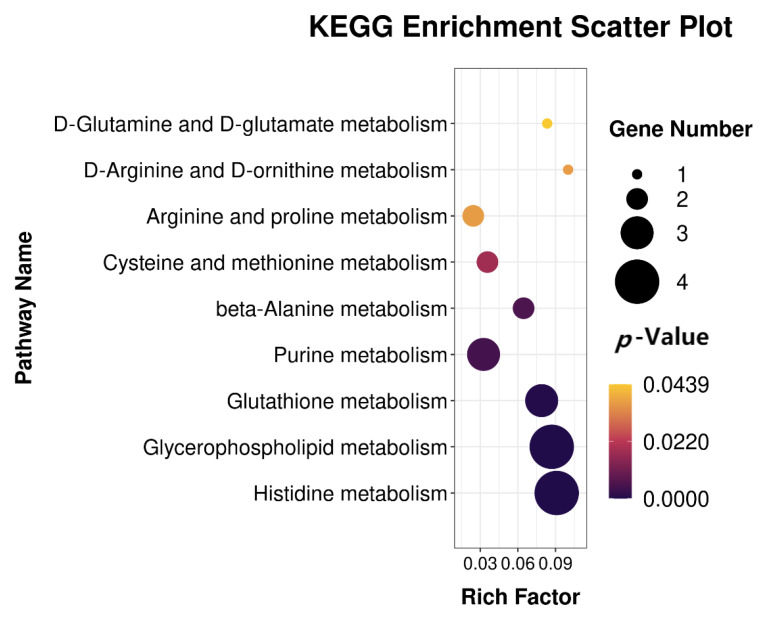
KEGG enrichment analysis of differential metabolites from MSTN-KO and WT cattle.

To further understand the biological function of the differential metabolites, KEGG functional annotation and pathway enrichment analysis of the differential metabolites was performed. Through the MBRole (http://csbg.cnb.csic.es/mbrole/, accessed on 11 October 2022) pathway analysis function, the KEGG ID of differential metabolites was used for pathway enrichment analysis. The results showed that 48 metabolic pathways were enriched from MSTN-KO and WT cattle. The top 10 metabolic pathways are shown in Table 6. Of these, nine metabolic pathways were significantly enriched, including glycerophospholipid metabolism, histidine metabolism, glutathione metabolism, purine metabolism, beta-alanine metabolism, cysteine, and methionine metabolism, D-arginine and D-ornithine metabolism, arginine and proline metabolism, and D-glutamine and D-glutamate metabolism (Figure 4). 

### 2.5. Association Analysis between Transcriptomic and Metabolomic Data

**Figure 5:**
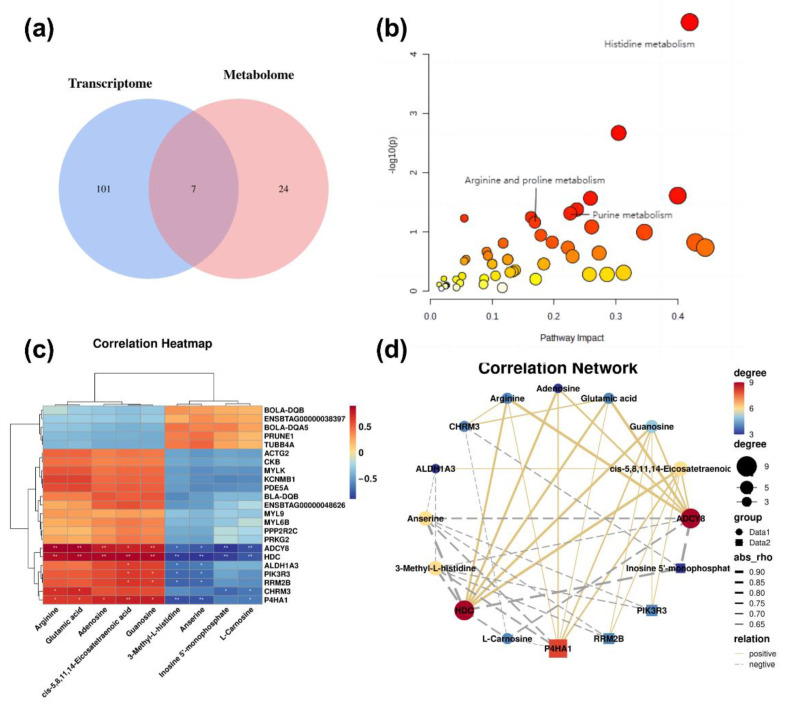
Integrated analysis of metabolomic and transcriptomic profiling of esophageal smooth muscle from MSTN-KO and WT cattle. (**a**) Venn diagram of shared KEGG terms among transcriptome and metabolome. (**b**) Integrated pathway analysis of transcriptomics and metabolomics. (**c**) Correlation heatmap of differentially expressed genes and metabolites for the integrated pathway. (**d**) Correlation network of differentially expressed genes and metabolites for the integrated pathway.

To investigate the association between differential metabolites and differentially expressed genes in esophageal smooth muscles of MSTN-KO and WT cattle, the KEGG pathway enrichment results were integrated. A Venn diagram plot indicated that differential metabolites and differentially expressed genes shared seven KEGG pathways (Figure 5a; Table 7). Of these, three pathways are associated with metabolism, mainly enriching in histidine metabolism, followed by purine metabolism, and then arginine and proline metabolism (Figure 5b). Moreover, according to the KEGG pathway enrichment analysis for the transcriptome and metabolome, we carried out a correlation test for metabolism-related differential metabolites and differentially expressed genes. Using the Pearson correlation analysis of 9 metabolites and 23 genes, a total of 7 genes were significantly positively or negatively correlated with one or more of 9 metabolites (Figure 5c,d).

## 3. Discussion

MSTN acts as a negative regulator of skeletal muscle growth and development, and its knockout affects skeletal muscle growth development and metabolism [32,33]. Recently, MSTN knockout has also been proven to have a role in smooth muscle growth and development [21,22,23]. In MSTN knockout pigs, increased penile corpus cavernosum smooth muscle area and smooth muscle-specific gene expression was found, indicating that MSTN knockout promoted smooth muscle growth [22]. Similarly, the esophageal smooth muscle area increased and muscle fiber types changed in MSTN knockout pigs [21]. Furthermore, Liu et al. found that MSTN knockout promoted the proliferation of uterine horn smooth muscle cells in MSTN knockout gilts [23]. In this study, the transcriptomic and metabolomic analysis found that MSTN knockout caused changes in the esophageal smooth muscle gene expression and metabolites. A combined transcriptome and metabolome analysis found that histidine metabolism, purine metabolism, and arginine and proline metabolism were enriched in the esophageal smooth muscle of MSTN knockout cattle. To the best of our knowledge, this is the first report on the effect of MSTN knockout on smooth muscle by transcriptomic and metabolomic analyses. These findings may provide research direction for exploring the specific mechanism of MSTN knockout affecting smooth muscle growth and development.

Histidine is a type of essential amino acid [34]. In addition to participating in protein metabolism, as a functional amino acid, it also has specific metabolic effects [35]. Histidine involves different metabolic pathways and can be methylated to 1-methyl or 3-methyl histidine, converted by transaminase to imidazole-pyruvic acid, condensed with β-alanine to form carnosine and anserine, or decarboxylated to form histamine [36]. Histidine metabolism was a significantly enriched pathway in the combined analysis of the metabolome and transcriptome of MSTN knockout bovine smooth muscle, involving differential metabolites including glutamic acid, 3-methyl-L-histidine, L-carnosine and anserine, and involving differentially expressed genes including ALDH3A1 and HDC. These results suggest that MSTN knockout regulates histidine metabolism in smooth muscles. It is well known that histamine, the product of histidine metabolism, plays an important role in regulating smooth muscle contraction [37]. This suggests that MSTN knockout may affect smooth muscle function, but needs to be evaluated in further studies.

Purine metabolism, the metabolic pathway that synthesizes and breaks down purines, is involved in diverse cellular processes such as energy storage, synthesis of nucleic acids and coenzymes, translation, and signaling [38,39]. The purines are a class of organic molecules that contain adenine-based derivatives (e.g., ATP, ADP, AMP, cAMP, NAD, adenosine), guanine-based derivatives (e.g., GTP, GDP, GMP, cGMP, guanosine), and related metabolites (hypoxanthine, xanthine, and uric acid) [40]. MSTN knockout has been reported to regulate ATP production [41,42]. In the present study, we found that MSTN knockout affected smooth muscle purine metabolism, further supplementing data that MSTN regulates energy metabolism. Furthermore, whether MSTN knockout affects other types of purine molecules in addition to regulating ATP content needs further study.

We also observed significant changes in arginine and glutamate, which belong to the arginine and proline metabolism pathways. Both arginine and proline belong to the glutamate family of amino acids [43]. Glutamate is not only the metabolic precursor of proline but also the final product of proline and arginine degradation [44]. Moreover, proline is the main metabolite of arginine metabolism [45]. Interestingly, arginine biosynthesis and glutamate metabolism were enriched in the rumen, reticulum, and omasum of MSTN knockout [46]. This study further indicates that MSTN knockout plays a role in the metabolism of arginine and proline.

## 4. Materials and Methods

### 4.1. Ethics Statement 

All experimental procedures in this study were consistent with the National Research Council Guide for the Care and Use of Laboratory Animals. All protocols were approved by the Institutional Animal Care and Use Committee at Inner Mongolia University (approval number: IMU-CATTLE-2022-050).

### 4.2. Animals and Sample Collection

As in our previous report [16], we used CRISPR/Cas9 and somatic cell nuclear transfer to generate MSTN knockout Chinese Luxi Yellow cattle. A total of 10 cattle, 5 MSTN^−/−^ Chinese Luxi Yellow cattle (male) and 5 wild-type Chinese Luxi Yellow cattle (male) were used. The cattle were slaughtered at the age of 24 months and fasted for 24 h before slaughter. Slaughter started in the morning, and all cattle were slaughtered by exsanguination. The process of slaughter followed the national standard operating procedures (GB/T 19477-2018, Cattle Slaughtering, China). The esophagus smooth muscle was collected within 30 min after slaughter and cut into several pieces and quickly placed in liquid nitrogen, followed by storage at −80 °C until further use [47].

### 4.3. Experimental Design

Animals were divided into two groups (group MSTN-KO and group WT) of 5 bovines each, 24-month-old, and mean body weights (BWs) of 608.5 ± 17.62 kg and 528.25 ± 11.03 kg, respectively. The cattle were fed in Hohhot, China (111°85′ E, 40°55′ N, 1040 m above sea level). Each barn contained about 240 m^2^ of indoor space and 300 m^2^ of an exercise yard, which can keep 15~20 cattle. Cattle were kept in the same environment, and each animal could move freely indoors and outdoors without restraint. Each cowshed was equipped with a constant temperature (15 °C) automatic watering system, and all cattle were free to drink water. The total mixed ration (TMR) diet consisted of 70% silage, 10% hay/alfalfa, and 20% supplementary grain feed. The forage/concentrate ratio was 4:1. The supplemental grain feed (Inner Mongolia Meng Yuan Kang Feed Co., Ltd., Hohhot, China) contained maize, soybean meal, soy flour, DDGS (distillers dried grains with solubles), calcium carbonate, calcium hydrogen phosphate, sodium chloride, trace elements, and vitamins. 

### 4.4. Transcriptome Sequencing and Analysis

Total RNA was isolated from esophageal smooth muscle tissues from MSTN-KO and WT cattle. The total RNA was extracted using Trizol reagent (Invitrogen, CA, USA) following the manufacturer’s procedure. Then, the cleaved RNA fragments were reverse-transcribed to create the final cDNA library following the protocol for the mRNA Seq sample preparation kit (Illumina, San Diego, CA, USA). The average insert size for the paired-end libraries was 300 bp (±50 bp). With five biological replicates for each group, a total of ten libraries were constructed. We then performed the paired-end sequencing on an Illumina Novaseq™ 6000 at the (Illumina, San Diego, CA, USA) following the vendor’s recommended protocol. After removing reads containing the adapter and low-quality sequences, the resulting high-quality valid data were mapped to the bovine reference genome (btau4.0). The mapped fragments were standardized using the fragments per kilobases per million reads (FPKM) method. DEG between MT and WT cattle was identified by the DEG-seq software package applying the MA-plot-based method with random sampling (MARS) model methods. The *p*-value < 0.05 and the |log2 fold change| > 1 were considered to have significant expression abundance. All DEGs were mapped to terms in the KEGG database. The raw sequence data reported in this paper have been deposited in the Genome Sequence Archive in BIG Data Center, Beijing Institute of Genomics (BIG), Chinese Academy of Sciences, with the accession number CRA010239 that are publicly accessible at https://bigd.big.ac.cn/gsa/browse/CRA010239 (accessed on 30 March 2023).

### 4.5. Metabolomic Analysis

Metabolites were extracted from esophageal smooth muscle tissues from MSTN-KO and WT cattle. With five biological replicates for each group, a total of ten libraries were constructed. A total of 3 mL of methanol and 0.64 mL water were added to each gram of tissue samples and homogenized in an ice bath. The samples were centrifuged at 4 °C, 15,000× *g* for 10 min, and the lower organic layers (with lipophilic compounds) were transferred into separate vials for LC-MS (LC-Bio, Hangzhou, China) analysis. Metabolite separations were performed with ACQUITY UPLC HSS T3 column (100 mm × 2.1 mm, 1.8 m, Waters, Manchester, UK) to analyze the esophageal smooth muscle tissue samples. The mass spectrometer was operated in both positive and negative ion modes for the analysis. XCMS [48] XCMS [48], CAMERA [49], and the metaX [50] toolbox were used to convert the raw data files. Metabolites were identified using the KEGG and HMDB [51] (http://www.hmdb.ca/, accessed on 1 December 2022) metabolic databases. The potential metabolites were screened based on the variable importance in the projection (VIP) values and Student’s *t*-test. VIP  >  1 and *p*  <  0.05 were considered as statistically significant. Enrichment analysis on the differentially expressed genes was carried out by the Kyoto Encyclopedia of Genes and Genomes (KEGG) (https://www.omicshare.com/tools/, accessed on 11 January 2023). 

### 4.6. Conjoint Analysis of Metabolome and Transcriptome 

The joint analysis was carried out on the metabolome and transcriptome. Overlapping pathways between the transcriptome and metabolome were identified, and Venn diagrams were created using the online Venn tools (https://www.omicstudio.cn/tool, accessed on 11 December 2022). The integrated pathway-level analysis of transcriptomic and metabolomic data was conducted with the “Joint Pathway Analysis” module in MetaboAnalyst (version 4.0) [52]. To study the correlation between the genes and metabolites, the differential genes and metabolites screened from the metabolome and transcriptome were jointly analyzed according to the Pearson correlation analysis method, and the heatmap and network were plotted for visualization (https://www.omicstudio.cn/tool, accessed on 11 December 2022).

## 5. Conclusions

This study analyzed the metabolomes and transcriptomes of esophageal smooth muscle tissues from MSTN-KO and WT cattle. A total of 466 differential genes and 130 differential metabolites were identified. Functional enrichment analysis showed that the DEGs were mainly enriched in 67 signaling pathways and differential metabolites were mainly enriched in 31 pathways. The transcriptome and metabolome were combined to analyze the significant enrichment pathways, and three metabolically related pathways, including histidine metabolism, purine metabolism, and arginine and proline metabolism were clarified. In total, seven differential genes were significantly associated with nine differential metabolites in these pathways. Collectively, these results indicated that MSTN knockout could induce comprehensive alterations both at transcriptional and metabolic levels. This study provides new basic data for the effect of MSTN knockout on muscle. However, the specific mechanism underlying which MSTN knockout regulates the signaling pathway identified in this study needs to be further investigated.

## Figures and Tables

**Table 1:** Summary of transcriptome sequencing data.

	Sample	Raw Data		Valid Date		Valid Ratio (Reads)	Mapped Reads	Q20%	Q30%	GC Content%
		Read	Base	Read	Base					
MK	MK_SM_1	56,174,110	8.43 G	53,357,872	8.00 G	94.99	51,717,009 (96.92%)	99.98	97.80	55
	MK_SM_2	61,375,252	9.21 G	56,467,142	8.47 G	92.00	54,543,271 (96.59%)	99.98	97.80	53
	MK_SM_3	48,954,976	7.34 G	46,576,650	6.99 G	95.14	44,966,614 (96.54%)	99.98	97.52	55
	MK_SM_4	47,170,368	7.08 G	44,582,584	6.69 G	94.51	43,069,247 (96.61%)	99.98	97.63	53.50
	MK_SM_5	48,462,004	7.27 G	46,238,274	6.94 G	95.41	44,849,512 (97.00%)	99.98	97.95	53
WT	WT_SM_1	59,383,830	8.91 G	55,872,426	8.38 G	94.09	54,208,590 (97.02%)	99.98	97.69	53.50
	WT_SM_2	61,905,908	9.29 G	58,183,966	8.73 G	93.99	56,488,445 (97.09%)	99.98	97.74	52.50
	WT_SM_3	58,039,162	8.71 G	54,405,778	8.16 G	93.74	52,466,230 (96.44%)	99.98	97.86	52
	WT_SM_4	57,335,900	8.60 G	54,514,652	8.18 G	95.08	52,970,312 (97.17%)	99.98	97.61	52.50
	WT_SM_5	41,341,780	6.20 G	39,744,658	5.96 G	96.14	38,508,054 (96.89%)	99.98	97.75	52.50

MK, MSTN knockout Chinese Luxi Yellow cattle; WT, wild-type Chinese Luxi Yellow cattle; MK_SM_1-5, MSTN knockout Chinese Luxi Yellow cattle smooth muscle 1–5; WT_SM_1-5, wild-type Chinese Luxi Yellow cattle smooth muscle 1–5.

**Table 2:** Top 10 differential genes.

Gene Name	*p*-Value	Log2 FC	Regulation
ENSBTAG00000005146	4.95795 × 10^−63^	−6.205321499	down
GRB14	8.1387 × 10^−23^	1.821394148	up
ENSBTAG00000033252	9.26458 × 10^−15^	−1.633757199	down
PRSS2	3.19021 × 10^−14^	14.3133367	up
BOLA-DQB	2.02928 × 10^−13^	−11.41895795	down
ENSBTAG00000054045	2.14561 × 10^−12^	−13.78520376	down
TPM1	2.97093 × 10^−12^	−1.096697574	down
PHTF1	8.66165 × 10^−12^	1.404694988	up
ACTN3	5.40909 × 10^−11^	−1.223344485	down
KRT75	1.02098 × 10^−9^	−2.809575033	down

**Table 3:** Top 10 GO terms.

GO ID	GO Term	GO Category	Q-Value
GO:0005615	extracellular space	Cellular Component	3.91405 × 10^−9^
GO:0005576	extracellular region	Cellular Component	1.38529 × 10^−6^
GO:0055010	ventricular cardiac muscle tissue morphogenesis	Biological Process	1.83908 × 10^−5^
GO:0014883	transition between fast and slow fiber	Biological Process	5.20773 × 10^−5^
GO:0005509	calcium ion binding	Molecular Function	5.20773 × 10^−5^
GO:0005887	integral component of the plasma membrane	Cellular Component	0.000143987
GO:0005886	plasma membrane	Cellular Component	0.001620284
GO:0097512	cardiac myofibril	Cellular Component	0.00192043
GO:0098978	glutamatergic synapse	Cellular Component	0.00255813
GO:0016020	membrane	Cellular Component	0.005612478

**Table 4:** Top 10 KEGG pathways.

Pathway ID	Pathway Name	Q-Value
bta04514	Cell adhesion molecules	1.16768 × 10^−7^
bta04261	Adrenergic signaling in cardiomyocytes	5.81554 × 10^−7^
bta05416	Viral myocarditis	1.86876 × 10^−6^
bta04260	Cardiac muscle contraction	2.56598 × 10^−6^
bta04612	Antigen processing and presentation	3.23385 × 10^−6^
bta04940	Type I diabetes mellitus	3.23385 × 10^−6^
bta05169	Epstein–Barr virus infection	1.13874 × 10^−5^
bta05332	Graft-versus-host disease	1.86997 × 10^−5^
bta05330	Allograft rejection	2.53395 × 10^−5^
bta05320	Autoimmune thyroid disease	4.24502 × 10^−5^

**Table 5:** The top ten differential metabolites of MSTN-KO and WT cattle.

Metabolite	KEGG ID	Log2 FC	VIP	*p*-Value
Glutamic acid	C19670	−2.294068752	42.55932058	2.43 × 10^−5^
Geranylcitronellol	null	−1.49330268	12.73319428	1.46 × 10^−5^
Palmitamide	NA	−1.107149268	9.77579363	8.89 × 10^−5^
Acetyl-DL-carnitine	NA	1.349414731	9.003102831	0.001746422
Stearamide	C13846	−1.317560034	7.297357056	0.002100988
L-Carnitine	C00318	1.416771295	6.926858962	0.001779882
L-Glutathione, reduced	C00051	−2.192035897	6.101367138	0.001271132
LysoPC 16:0	C04230	−1.878247824	4.295401189	0.000139281
cis-5,8,11,14-Eicosatetraenoic acid	C00219	−2.805619747	4.076439099	0.000268185
Isopropyl tiglate	null	1.860302559	3.83487271	0.000437783

**Table 6:** Top 10 metabolic pathways.

Pathway ID	Pathway Name	*p*-Value
map00564	Glycerophospholipid metabolism	1.97 × 10^−5^
map00340	Histidine metabolism	1.64 × 10^−5^
map00970	Aminoacyl-tRNA biosynthesis	0.000136979
map01100	Metabolic pathways	0.000204554
map02010	ABC transporters	0.000278192
map00480	Glutathione metabolism	0.000335696
map04730	Long-term depression	0.00046444
map05014	Amyotrophic lateral sclerosis (ALS)	0.000579276
map04742	Taste transduction	0.000997487
map01064	Biosynthesis of alkaloids derived from ornithine, lysine, and nicotinic acid	0.001782761

**Table 7:** Shared KEGG pathway between the transcriptome and metabolome.

KEGG Pathway	KEGG ID
Vascular smooth muscle contraction	04270
Leishmaniasis	05140
Histidine metabolism	00340
Purine metabolism	00230
Gap junction	04540
Arginine and proline metabolism	00330
Chagas disease	05142

## Data Availability

The data presented in this study are available on request from the corresponding author.

## Supplementary Materials

The following supporting information can be downloaded at: https://www.mdpi.com/article/10.3390/ijms24098120/s1.
